# Reliability and validity analysis of personality assessment model based on gait video

**DOI:** 10.3389/fnbeh.2022.901568

**Published:** 2022-08-02

**Authors:** Yeye Wen, Baobin Li, Deyuan Chen, Tingshao Zhu

**Affiliations:** ^1^School of Electronic, Electrical and Communication Engineering, University of Chinese Academy of Sciences, Beijing, China; ^2^Institute of Psychology, Chinese Academy of Sciences, Beijing, China; ^3^School of Computer Science and Technology, University of Chinese Academy of Sciences, Beijing, China; ^4^Department of Psychology, University of Chinese Academy of Sciences, Beijing, China

**Keywords:** personality assessment, gait video, big data, machine learning, reliability and validity

## Abstract

Personality affects an individual’s academic achievements, occupational tendencies, marriage quality and physical health, so more convenient and objective personality assessment methods are needed. Gait is a natural, stable, and easy-to-observe body movement that is closely related to personality. The purpose of this paper is to propose a personality assessment model based on gait video and evaluate the reliability and validity of the multidimensional model. This study recruited 152 participants and used cameras to record their gait videos. Each participant completed a 44-item Big Five Inventory (BFI-44) assessment. We constructed diverse static and dynamic time-frequency features based on gait skeleton coordinates, interframe differences, distances between joints, angles between joints, and wavelet decomposition coefficient arrays. We established multidimensional personality trait assessment models through machine learning algorithms and evaluated the criterion validity, split-half reliability, convergent validity, and discriminant validity of these models. The results showed that the reliability and validity of the Gaussian process regression (GPR) and linear regression (LR) models were best. The mean values of their criterion validity were 0.478 and 0.508, respectively, and the mean values of their split-half reliability were all greater than 0.8. In the formed multitrait-multimethod matrix, these methods also had higher convergent and discriminative validity. The proposed approach shows that gait video can be effectively used to evaluate personality traits, providing a new idea for the formation of convenient and non-invasive personality assessment methods.

## Introduction

Personality is a characteristic set of behavior, cognition and psychological state, with stability and persistence (Corr and Matthews, 2020). Personality affects the behavior, mental states and subjective well-being of individuals (Costa and McCrae, 1980; Baumert et al., 2017). Studies have shown that personality traits are even related to physical health factors such as obesity (Jokela et al., 2013) and the risk of death (Iwasa et al., 2008). In addition, personality is closely related to academic achievements and learning styles (Komarraju et al., 2011), career choices and satisfaction (Seibert et al., 2001; Kern et al., 2019), love quality and marriage relationships (Holland and Roisman, 2008). Based on these issues, the need for a more convenient and objective user personality evaluation approach has become increasingly urgent.

Currently, many personality assessment methods are available, and questionnaires and scales are the most widely used measurement tools (Bing et al., 2007; Rammstedt and John, 2007). However, self-reported questionnaires are not applicable in some occasions. For example, in the psychological assessment of job hunting or enrolment, the participants may have more motives for answering deceptively when interests are involved (Ones et al., 1996). In addition, questionnaires are not suitable for situations where multiple measurements are required, because filling out the same questionnaire multiple times leads to practice effects.

As a natural and easily observed body movement, human gait conveys much information about emotions, cognition, intentions, and personality (Matsumoto et al., 2015). Previous studies have shown that it is possible to establish a relationship between certain qualities of body motion and personality (Koppensteiner and Grammer, 2010). The walking speed of a person in adulthood reflects, in part, the individual’s personality (Stephan et al., 2018). Higher degrees of extroversion and conscientiousness are associated with faster initial walking speeds and lesser walking speed declines, while high neuroticism is manifested by slow walking (Tolea et al., 2012; Agmon and Armon, 2016). Hand movements can also effectively express personality traits (Wang et al., 2016). For instance, the more open a person is, the more violent their vertical arm movements and the more obvious the changes in their movement directions (Koppensteiner and Grammer, 2010). In addition, thoracic and pelvic movements and the coordination of limbs are related to personality (Satchell et al., 2017). For example, individuals with high neuroticism and low extroversion show decreased mobility and poor limb coordination (LeMonda et al., 2015).

Although much evidence has shown that personality can be reflected by gait, a personality assessment method based on gait has not yet been fully established. Previous research has mainly focused on the statistical correlations between personality traits and gait. Sun et al. (2019) performed preliminary research explorations regarding the modeling of gait and personality traits, but they lacked a comprehensive evaluation of the model’s performance. Since personality contains many traits, it is necessary to establish a multidimensional model. Existing model evaluation methods, such as accuracy- or error-based approaches, cannot evaluate the correlations between the dimensions of the model. Therefore, we apply the reliability and validity evaluation method used for scales to a machine learning model. We use the correlations between the prediction scores of each dimension of personality obtained from models and actual scores from scales to calculate the model validity and use the correlations between the predicted scores of models based on the two halves of the input gait data to calculate the model reliability. This method has been proven feasible in the field of affective computing (Park et al., 2015).

In addition, a practical method should also include convenient tools to record gait data. The need for expensive and complex facilities in previous studies, such as motion capture systems (Mündermann et al., 2006; Leardini et al., 2007; O’Connor et al., 2007), smart wearable devices (Tao et al., 2012), and Kinect (Springer and Yogev Seligmann, 2016; Sun et al., 2018), made them unusable as real-life solutions. In real life, due to the popularity of cameras, we can easily obtain gait video. Many studies have shown that gait video data can be used to achieve efficient gait recognition (Singh et al., 2018; Wan et al., 2018), providing ideas for us to use gait video to establish a personality assessment model. Because gait video is convenient, easy to obtain and non-invasive, we can carry out large-scale gait experiments.

This study uses ordinary cameras to record two-dimensional gait video, builds a multidimensional machine learning model, and explores the criterion validity, split-half reliability, convergent validity, and discriminant validity of the developed personality assessment model. The purpose is to provide a new convenient personality assessment approach.

## Materials and methods

### Data collection

#### Participants

The personalities of adult individuals tend to be stable (Briley and Tucker-Drob, 2014). For meaningful evaluation, datasets should contain at least 30 participants and possibly more (Hofmann et al., 2014). We recruited 152 adult participants without mental illness or physical disability, including 79 males (52%) and 73 females (48%) with an average age of approximately 23 years (SD = 1.07).

#### Collection process

**FIGURE 1:**
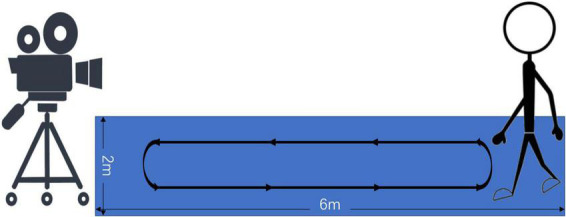
Experimental setup for gait data collection. The camera was fixed on the side of a rectangular footpath with a size of 6 m × 2 m. Participants walked back and forth on the footpath.

Participants walked back and forth for 2 min in a rectangular area with a size of 6 m × 2 m according to their daily walking conditions. During this period, a camera was used to record the participants’ gait videos. The experimental setup for gait data collection is shown in Figure 1.

After completing the gait collection process, the participants immediately filled out the Big Five Inventory (BFI-44) personality scale. The BFI consists of 44 items and five subscales: extraversion (8 items), agreeableness (9 items), conscientiousness (9 items), neuroticism (8 items), and openness (10 items) (John et al., 1991). Each item of the BFI-44 is assessed on a five-point Likert scale, ranging from 1 (“disagree strongly”) to 5 (“agree strongly”). This study used the Chinese version of the BFI-44 scale. The range of Cronbach’s alpha was 0.698–0.807, and the test-retest reliability was between 0.694 and 0.770 (Carciofo et al., 2016).

The above protocol was performed with permission from the Institutional Review Board of the Institute of Psychology, Chinese Academy of Sciences (approval number: H15010).

### Data preprocessing

#### Key point extraction

**FIGURE 2:**
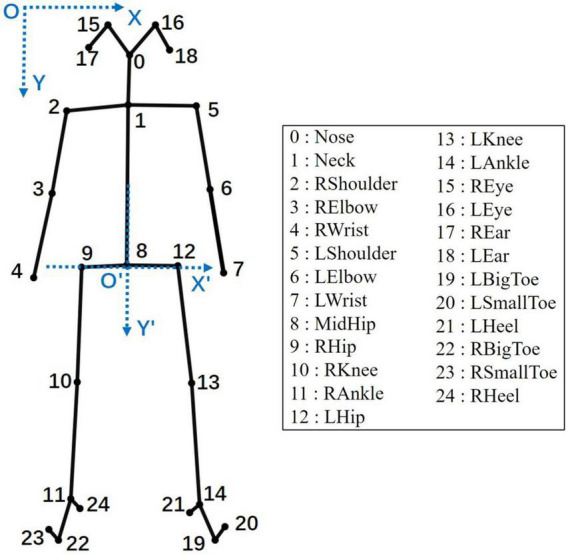
The 25 key points of the human body in OpenPose. In the key point labels, L and R represent the left and right sides of the human skeleton, respectively. The *XOY* in the upper left is the original coordinate system, and the *X*′*O*′*Y*′ in the middle is the new coordinate system formed after coordinate translation.

We used OpenPose to extract skeleton coordinates from the gait videos. OpenPose is a human posture recognition system that can detect key points of human body, hands, face, and feet (Cao et al., 2021). This study used OpenPose to extract the two-dimensional coordinates of 25 key points of the body, as shown in Figure 2.

#### Data unification

The experimental setup in Figure 1 involved walking back and forth, so the gait video contained the participants’ front and back gaits. Related studies have shown that gait skeleton evaluation based on the front view is more accurate than that based on the back view (Fang et al., 2019). The differences in the amounts of training data available for the participants can greatly affect the performance of machine learning models (Luyckx and Daelemans, 2010). Therefore, we kept at least four complete gait cycles based on the front view for each participant. One gait cycle represented the process of one foot from leaving the ground until landing (Baker and Hart, 2013). In this study, the gait frames of all participants were unified to 75 frames. This method has been used in many studies and has proven effective (Sun et al., 2017; Zhao et al., 2019; Yeye et al., 2020).

#### Coordinate translation

**FIGURE 3:**
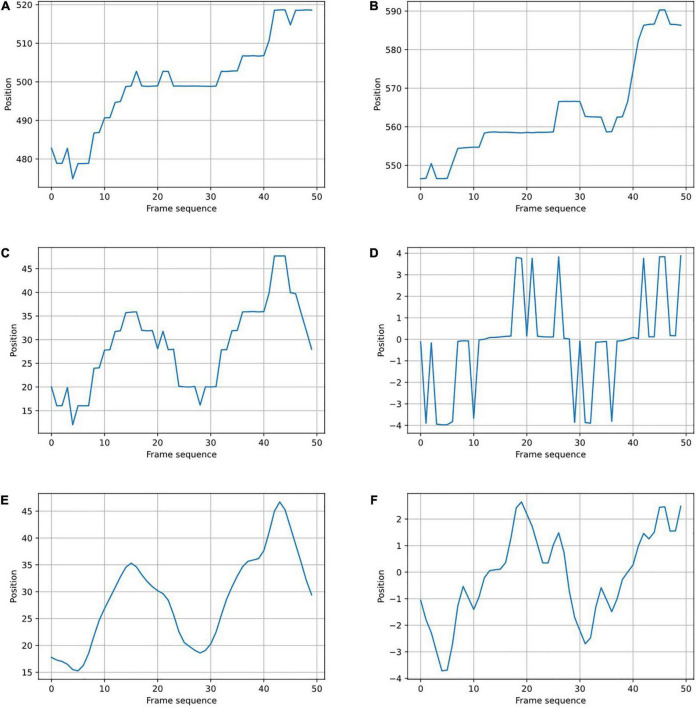
Coordinate sequence of key points of gait. **(A,B)** Before coordinate translation, in the *XOY* coordinate system. **(C,D)** After coordinate translation, before filtering, in the *X*′*O*′*Y*′ coordinate system. **(E,F)** After filtering. The left figures show the x-coordinate sequence of the LAnkle key point. The right figures show the y-coordinate sequence of the RHip key point.

In the *XOY* original coordinate system (Figure 2), the coordinates of key points were greatly affected by the body shapes and positions of the participants, and their coordinate sequences changed irregularly, as shown in A and B of Figure 3. The movement of the human center of gravity can be used to assess the stability of a person’s gait (Iida and Yamamuro, 1987). The movement changes between the center of gravity and the center of the pelvis are very similar, and the movement of the pelvis during the gait is obviously related to the movements of the limbs and torso (Whittle, 1997). Therefore, this study used the key points of MidHip (No. 8) as the coordinate origin to establish a new coordinate system *X*′*O*′*Y*′ (Figure 2). The coordinate translation formula is as follows:


$$\left\{ \begin{array}{c} x_{i}^{\prime }={\text{x}}_{i}-{\text{x}}_{8}\\ {\text{y}}_{i}^{\prime }={\text{y}}_{i}-{\text{y}}_{8} \end{array}\right.$$

where *i* = 1,2,…,24 and *x_i_* and *y_i_* represent the horizontal and vertical coordinates of point i in the *XOY* coordinate system, respectively. After coordinate translation, the coordinate sequence obeyed an obvious motion law, reflecting the periodicity of gait movement, as shown in C and D of Figure 3.

#### Filtering

**FIGURE 4:**
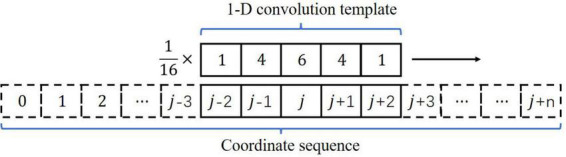
Filtering. The one-dimensional convolution kernel was [1,4,6,4,1].

Due to the interference of the video background, high-frequency noise was contained in the key point coordinates, as shown in Figure 3. We used a template with a one-dimensional convolution kernel ([1,4,6,4,1]) to smooth the coordinate sequence, as shown in Figure 4. The filtering formula is:


$${\text{j}}^{\prime }=\frac{1}{16}\times [\left(\text{j}-2\right)\times 1+\left(\text{j}-1\right)\;\times 4+\text{j}\times 6+\left(\text{j}+1\right)\times 4+\left(\text{j}+2\right)\times 1]$$

where *j* represents the coordinate value of the jth frame before filtering, and *j*′ represents the coordinate value after filtering. The time series data after filtering (E and F in Figure 3) were obviously smoother than the original data (C and D in Figure 3).

### Feature engineering

#### Feature construction

##### Interframe difference

Gait is the dynamic change of the body exhibited while walking (Minetti, 1998). The movement information of a gait is contained in the frame-to-frame changes shown in the corresponding gait video. We used the forward interframe difference method to focus on the key point coordinate changes between two adjacent frames. The forward difference formula is:


$$△\,{\text{f}}_{j}={\text{f}}_{j+1}-{\text{f}}_{j}$$

where *f*_*j+1*_ and *f_j_* represent the skeleton coordinates of the (j+1)th frame and the jth frame, respectively, and △*f*_*j*_ represents the difference between two adjacent frames.

##### Distance between joints

Gait requires coordinated movement among the joints of the body (Murray, 1967; Luyckx and Daelemans, 2010). Therefore, gait research cannot examine the movement rules of a certain key point or joint in isolation. The distance between a pair of joints is a local motion unit composed of two joint points. We used the horizontal and vertical distances between joints to characterize the changes between two joint points. We found that the distance between joints had a corresponding meaning in terms of gait movement. For example, the distance between LWrist (No. 7) and RWrist (No. 4) represented the swing of the hands at the associated moment (Donker et al., 2002; Park, 2008), and the distance between LAnkle (No. 14) and RAnkle (No. 11) represented the stride at that moment (Whittle, 2014). We proposed 13 distances between joints, including 26 coordinate distances. See Table 1 for specific indicators.

**TABLE 1:** Distances between joints.

Name*	Meaning	Name*	Meaning
dist_1_0_x	Head swing	dist_10_9_x	Thigh swing
dist_1_0_y		dist_10_9_y	(right)
dist_3_2_x	Upper arm	dist_11_9_x	Leg swing
dist_3_2_y	swing (right)	dist_11_9_y	(right)
dist_4_2_x	Arm swing	dist_13_12_x	Thigh swing
dist_4_2_y	(right)	dist_13_12_y	(left)
dist_6_5_x	Upper arm	dist_14_12_x	Leg swing
dist_6_5_y	swing (left)	dist_14_12_y	(left)
dist_7_5_x	Arm swing	dist_13_10_x	Relative swing
dist_7_5_y	(left)	dist_13_10_y	of both knees
dist_6_3_x	Relative swing	dist_14_11_x	Relative swing
dist_6_3_y	of both elbows	dist_14_11_y	of both feet
dist_7_4_x	Relative swing		
dist_7_4_y	of both hands		

*In the name, dist_a_b_x and dist_a_b_y, respectively, represent the x-axis distance and the y-axis distance between joints a and b, where a and b are key point (or joint) labels.

##### Angle between joints

**FIGURE 5:**
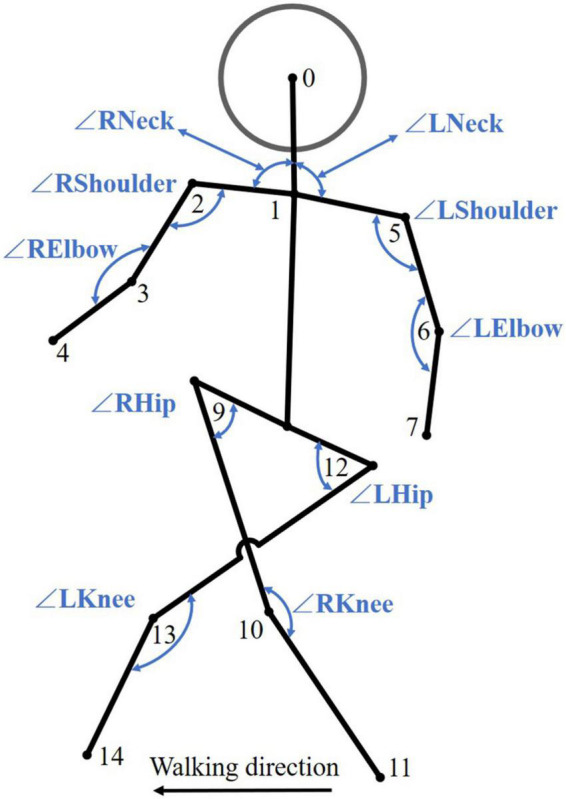
Angle between the joints of the human body. Some unused key points have been omitted.

The angle between joints is also an important indicator for measuring human gait movement (Davis et al., 1991). The angle between joints is a local motion unit composed of multiple joints. We used the angle formed by 3 joint points to characterize the relative motion between multiple joints. For example, the angle between Nose (No. 0), Neck (No. 1), and RShoulder (No. 2) represented the tilt of the head at the associated moment, and the angle between RHip (No. 9), RKnee (No. 10), and RAnkle (No. 11) represented the bending movement of the right knee at this moment (Seel et al., 2014). We proposed 10 angles between joints, as shown in Figure 5. See Table 2 for specific indicators. It is worth noting that ∠*RNeck* and ∠*LNeck* both represented the tilt angle of the neck. However, when participants shrugged or slanted their shoulders, the two angles did not constitute a supplementary angle. As MidHip (No. 8) was eliminated during preprocessing, for ∠*LHip* and ∠*RHip*, we used *angle*_9_12_13 and *angle*_12_9_10 instead of *angle*_8_12_13 and *angle*_8_9_10, respectively.

**TABLE 2:** Angles between joints.

Name*	Meaning	Symbol
angle_0_1_2	Neck angle (right)	∠RNeck
angle_0_1_5	Neck angle (left)	∠LNeck
angle_1_2_3	Shoulder angle (right)	∠RShoulder
angle_1_5_6	Shoulder angle (left)	∠LShoulder
angle_2_3_4	Elbow angle (right)	∠RElbow
angle_5_6_7	Elbow angle (left)	∠LElbow
angle_12_9_10	Hip angle (right)	∠RHip
angle_9_12_13	Hip angle (left)	∠LHip
angle_9_10_11	Knee angle (right)	∠RKnee
angle_12_13_14	Knee angle (left)	∠LKnee

*In the name, angle_a_b_c represents the ∠abc composed of joints a, b, and c, where a, b, and c are key points (or joints) labels.

##### Wavelet transform

In frequency domain analysis, the Fourier transform is often used to observe signal spectra, but this transform is not suitable for analyzing signals whose frequencies change with time (Oppenheim et al., 1996). The short-time Fourier transform developed on this basis realizes time-frequency localization by adding a moving window function, but a problem remains: the window function cannot change with the frequency (Kwok and Jones, 2000). The wavelet transform overcomes the above shortcomings and can realize multiresolution analysis (Daubechies, 1992). In the gait video, through observing the raw gait data, we found that there are differences in the movement amplitude and frequency of different key points. For example, RWrist (No. 4) has a larger movement amplitude than REye (No. 17), but REye has a faster movement frequency. In order to express the difference in frequency, we chose the wavelet transform and used the “haar” wavelet basis to decompose the gait skeleton coordinate sequence with 5 layers of wavelets. The source signal *X* is decomposed into:


$$\text{X}={\text{D}}_{1}+{\text{D}}_{2}+{\text{D}}_{3}+{\text{D}}_{4}+{\text{D}}_{5}+{\text{A}}_{5}$$

where *D_1_*, *D_2_*, *D_3_*, *D_4_*, and *D_5_* are the high-frequency signals (or detail coefficient arrays) decomposed from the first to fifth layers, respectively, and *A_5_* denotes the low-frequency signal (or approximate coefficient array) decomposed from the fifth layer.

#### Feature extraction

**FIGURE 6:**
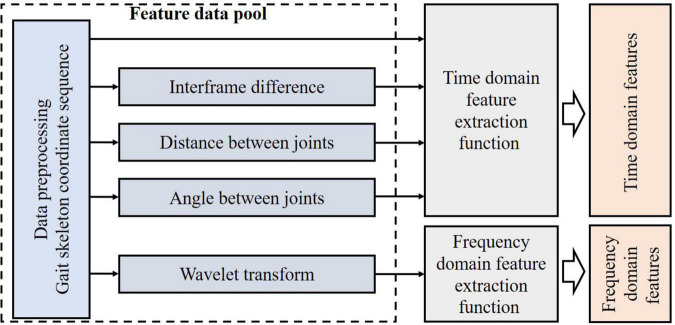
Flow chart of feature extraction.

For feature construction, we constructed a feature data pool, including gait skeleton coordinates, interframe differences, distances between joints, angles between joints, and frequency domain arrays of wavelet decomposition (left side of Figure 6). We extracted the time-frequency domain features based on the feature data pool. In Figure 6, we used different colors to distinguish the changes of data. Different colors from left to right represented the original coordinate sequences, feature construction, feature extraction functions and extracted features, respectively.

##### Time domain feature extraction

Since the gaits included both simple linear motions and complex non-linear processes (Iqbal et al., 2015), we used 10 linear and non-linear functions for time domain feature extraction, as shown in Table 3. Based on the time series data in the feature data pool, we obtained 1320-dimensional time domain features.

**TABLE 3:** Time domain feature extraction functions.

Feature extraction function*	Meaning
maximum (x)	The maximum value of x.
minimum (x)	The minimum value of x.
mean (x)	The mean value of x.
median (x)	The median of x.
variance (x)	The variance of x.
root_mean_square (x)	The root mean square of x.
skewness (x)	The skewness of x.
kurtosis (x)	The kurtosis of x.
abs_energy (x)	The absolute energy of x.
variation_coefficient (x)	The coefficient of variation of x.

*x represents time series data.

##### Frequency domain feature extraction

Due to the complexity of gait, some gait patterns cannot be distinguished in the time domain, but some laws can be reflected in the frequency domain (Orović et al., 2011). We calculated the absolute maximum values, mean values, variances, and absolute energy of the 6 coefficient arrays after wavelet decomposition in the feature data pool and obtained 1152-dimensional frequency domain features.

### Modeling

Compared with the number of participants, the dimensionality of the 2472-dimensional time-frequency features obtained after feature extraction was too high, and this easily led to serious model overfitting (Bishop, 2006). We used principal component analysis (PCA) to reduce the dimensionality of the standardized features. Then we used Sequential Forward Selection (SFS) to select 40 features accordingly based on the properties of different machine learning algorithms in the modeling. SFS added features to form a feature subset in a greedy fashion. At each stage, the estimator (machine learning algorithm) chose the best feature to add based on the cross-validation score of an estimator. While selecting features, the performance of the model was continuously improved. Finally, SFS selected a feature subset containing 40 features for each model. This subset enabled the model to achieve optimal performance.

Personality assessment is a regression task. Different regression algorithms have different characteristics. Linear regression (LR) is often used as a baseline model because of its fast calculation speed, low complexity and easy interpretation. Gaussian process regression (GPR) is widely used in time series analysis, and gait videos are time series data. Random forest regression (RFR) is a typical ensemble algorithm, which can deal with errors caused by imbalanced gait data. Support vector regression (SVR) is effective in high-dimensional gait time-frequency feature space. Therefore, we selected 7 typical machine learning regression algorithms for modeling, namely GPR, LR, RFR, SVR, where the SVR algorithm contains 4 kernel functions: “linear,” “poly,” “rbf,” and “sigmoid.” The kernel function directly determines the final performance of the SVR algorithm, but the selection of an appropriate kernel function has always been an unsolved problem (Zhou, 2021), so we made 4 attempts with the SVR algorithm.

**FIGURE 7:**
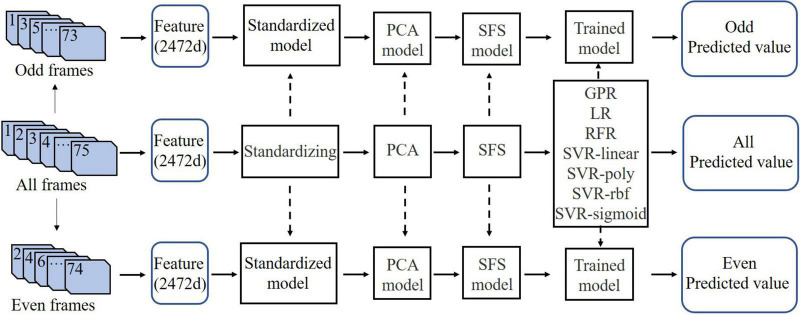
Modeling flowchart. The solid arrows indicate the data flows, and the dotted arrows indicate the applications of the model. “Odd frames” and “even frames” were divided from the first 74 frames among “all frames.” PCA, principal component analysis; SFS, sequential forward selection; GPR, Gaussian process regression; LR, linear regression; RFR, random forest regression. SVR-linear, SVR-poly, SVR-rbf, and SVR-sigmoid represent the SVR model using the “linear,” “poly,” “rbf,” and “sigmoid” kernel functions, respectively.

As shown in Figure 7, the modeling process included three data streams, which contained 75 frames of complete data and odd-even split-half data. We used “all frames” to train the standardization, PCA, SFS, and algorithm models and applied these models to “odd frames” and “even frames.”

### Evaluation

We trained and tested the model with 10 times of 10-fold cross validation and used criterion validity, odd-even split-half reliability, convergent validity, discriminant validity and root mean square error (RMSE) as the model evaluation indicators. RMSE is the most commonly used performance metric in regression tasks (Zhou, 2021) and is defined as:


$$\text{R}\,M\,S\,E=\sqrt{\frac{1}{\text{N}}\,\sum_{n=1}^{N}{\left(\text{P}\,r\,e\,d\,i\,c\,t\,e\,d_{n}-\text{A}\,c\,t\,u\,a\,l_{n}\right)}^{2}}$$

where *Predicted*_*n*_ and *Actual*_*n*_ represent the personality prediction score and personality scale score of the nth participant, respectively.

#### Criterion validity and split-half reliability

Using the BFI-44 scores as ground truth, criterion validity was assessed by examining the Pearson correlation coefficient between the model and scale scores for each personality trait. We chose the odd-even split-half reliability as the reliability indicator. The Pearson correlation coefficient between model prediction scores for the “odd frames” and “even frames” was used to evaluate the odd-even split-half reliability of the model. That is, the correlation coefficient between “odd predicted value” and “even predicted value” in Figure 7 was calculated to indicate reliability.

#### Convergent and discriminant validity

This study used a multitrait-multimethod matrix to explore the convergent and discriminant validity of the personality assessment models. The matrix was filled by Pearson correlation coefficients and included five personality traits (extraversion, agreeableness, conscientiousness, neuroticism, and openness) and two measurement methods (the BFI-44 scale and personality assessment model). Convergent validity was numerically the same as criterion validity. However, when evaluating convergent validity, the BFI-44 scores were no longer used as the calibration, but the BFI and the model were regarded as a method of measuring personality, respectively. Discriminant validity was assessed by comparing the magnitude of between-trait correlations (e.g., between extraversion and conscientiousness) within models with those within BFI-44.

#### Statistical analysis

To explore the relationships between the features used for modeling and the gait joint points, we mapped the optimal feature combination selected by SFS to 24 key points (MidHip was the coordinate origin and was eliminated during data preprocessing). For feature engineering, features were constructed based on the gait skeleton coordinates, so the mapping process was the inverse process of feature construction. In statistical analysis, we counted each key point with different weights according to the rules of feature construction, so as to avoid deviations in the results of statistical analysis due to different key points used in constructing features.

## Results

### Criterion validity and split-half reliability

In Table 4, r_1_ represents the criterion validity, and r_2_ represents the odd-even split-half reliability. Among the 7 algorithm models, the GPR and LR models had the best criterion validity (the mean r_1_ values were 0.478 and 0.508, respectively, and their RMSE values were lower than other algorithms), and r_1_ was above 0.4 for all personality traits. The other models exhibited unbalanced performance across different personality traits. In addition, except for that of the SVR-poly and RFR models, the split-half reliability of the other models was good, and the mean values of r_2_ were above 0.8. In general, the GPR and LR models had the best performance with good criterion validity and split-half reliability, as shown in Table 4. The results of the remaining models are listed in Supplementary Appendix A of the Supplementary Materials. (The appendices are in the Supplementary Materials, the same below).

**TABLE 4:** Criterion validity and split-half reliability of the GPR and the LR personality assessment models.

	Gaussian process regression			Linear regression		
		
	RMSE	*r*_1_	*r*_2_	RMSE	*r*_1_	*r*_2_
Extraversion	5.416	0.442	0.834	5.512	0.427	0.831
Agreeableness	4.690	0.405	0.840	4.550	0.487	0.921
Conscientiousness	6.015	0.464	0.900	6.000	0.474	0.870
Neuroticism	5.666	0.580	0.861	5.711	0.580	0.858
Openness	5.435	0.500	0.770	5.305	0.574	0.802

RMSE, root mean squared error. r_1 represents the criterion validity, and r_2 represents the split-half reliability. All correlation coefficients are highly significant (p < 0.001).

### Convergent and discriminant validity

In the multitrait-multimethod matrix (as shown in Tables 5, 6), the bold numbers represent the correlations between different methods for measuring the same trait, the italic numbers represent the correlations between different traits measured by the same method, and the numbers in the rectangular area (except those in bold) represent the correlations between different methods for measuring different traits.

**TABLE 5:** Convergent and discriminant validity of the GPR personality assessment model (GPR-PAM).

	GPR-PAM					BFI-44				
		
	E	A	C	N	O	E	A	C	N	O
**GPR-PAM**										
E										
A	*0.154*									
C	*0.223*	*0.220*								
N	*−0.339*	*−0.329*	*−0.277*							
O	*0.146*	*0.220*	*0.244*	*−0.298*						
**BFI-44**										
E	**0.442**	0.082	0.155	−0.241	0.100					
A	0.252	**0.405**	0.187	−0.339	0.213	*0.415*				
C	0.241	0.209	**0.464**	−0.321	0.276	*0.454*	*0.523*			
N	−0.301	–0.222	−0.243	**0.580**	−0.281	*−0.596*	*−0.678*	*−0.584*		
O	0.257	0.232	0.279	−0.255	**0.500**	*0.273*	*0.220*	*0.448*	*−0.285*	

E, extraversion; A, agreeableness; C, conscientiousness; N, neuroticism; O, openness. All correlation coefficients are highly significant (p < 0.001). Bold numbers represent the correlations between GPR-PAM and BFI-44 for measuring the same trait, the italic numbers represent the correlations between different traits measured by GPR-PAM or BFI-44.

**TABLE 6:** Convergent and discriminant validity of the LR personality assessment model (LR-PAM).

	LR-PAM					BFI-44				
		
	E	A	C	N	O	E	A	C	N	O
**LR-PAM**										
E										
A	*0.214*									
C	*0.225*	*0.166*								
N	*−0.362*	*−0.457*	*−0.293*							
O	*0.159*	*0.116*	*0.240*	*−0.280*						
**BFI-44**										
E	**0.427**	0.118	0.192	−0.240	0.116					
A	0.256	**0.487**	0.187	−0.339	0.185	*0.415*				
C	0.235	0.200	**0.474**	−0.320	0.288	*0.454*	*0.523*			
N	−0.331	–0.335	−0.268	**0.580**	−0.260	*−0.596*	*−0.678*	*−0.584*		
O	0.227	0.187	0.268	−0.254	**0.574**	*0.273*	*0.220*	*0.448*	*−0.285*	

E, extraversion; A, agreeableness; C, conscientiousness; N, neuroticism; O, openness. All correlation coefficients are highly significant (p < 0.001). Bold numbers represent the correlations between LR-PAM and BFI-44 for measuring the same trait, the italic numbers represent the correlations between different traits measured by LR-PAM or BFI-44.

Among all the models, the average convergence correlation of the optimal LR model was 0.508, and the mono-trait Pearson correlations between the assessment methods were extraversion: *r* = 0.427; agreeableness: *r* = 0.487; conscientiousness: *r* = 0.474; neuroticism: *r* = 0.580; and openness: *r* = 0.574 (Table 6). The average convergence correlation of the GPR model (*r*_*GPR*_*BFI*_ = 0.478, see Table 5) was close to that of the LR model, while that of the other models is poor (*r*_*RFR*_*BFI*_ = 0.145, *r*_*SVRlinear*_*BFI*_ = 0.137, *r*_*SVRpoly*_*BFI*_ = 0.286, *r*_*SVRrbf*_*BFI*_ = 0.333, *r*_*SVRsigmoid*_*BFI*_ = 0.316, see Supplementary Appendix B). In LR and GPR models, the bold numbers were significantly larger (*p* < 0.001) than the values in the same column or row of the rectangular area, which showed that our models had good convergent validity.

The discriminant validity coefficients for each method were shown in italics. The average magnitudes (absolute value; the same below) of the discriminant validity coefficient of models were significantly lower than that of the BFI-44 scale (*r*_*GPR*_ = 0.245, *r*_*LR*_ = 0.251, *r*_*RFR*_ = 0.069, *r*_*SVRlinear*_ = 0.050, *r*_*SVRpoly*_ = 0.093, *r*_*SVRrbf*_ = 0.164, *r*_*SVRsigmoid*_ = 0.098, in the upper left triangle; *r*_*BFI*_ = 0.448, in the lower right triangle; *p* < 0.001) in Tables 5, 6 and Supplementary Appendix B. Because italics indicated the correlation between different traits, a small correlation coefficient indicated good discriminant validity. This showed that the models were relatively better than the BFI-44 at discriminating between traits. In addition, the average magnitudes of the convergent validity coefficients of the models were significantly greater than the average magnitudes of their discriminant validity coefficients (*r*_*Model*_*BFI*_ = *r*_*Model*_, *p* < 0.001), indicating the models had good discriminant validity. However, the RFR, SVR-linear, SVR-poly, SVR-rbf, and SVR-sigmoid models had poor convergent validity, that is, poor predictive performance, which may lead to large deviations in the above discriminant validity. In summary, the LR and GPR models had relatively good convergent and discriminant validity.

### Statistical analysis

**FIGURE 8:**
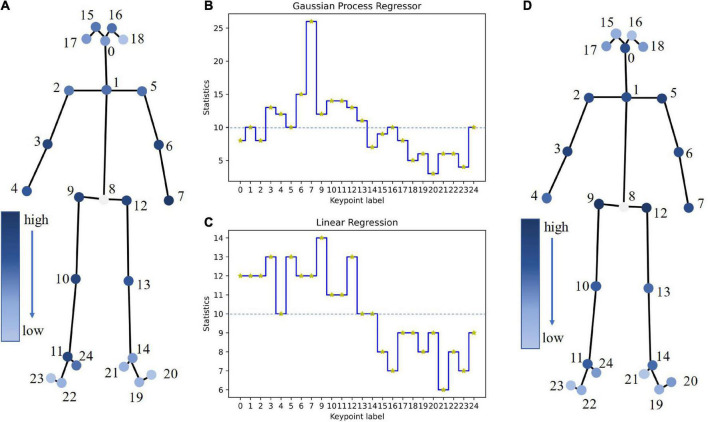
Feature and key point mapping. **(B,C)** Statistical ladder diagrams of the GPR and LR models. The horizontal axis represents the labels of the key points, the vertical axis represents the statistical values of the key points, and the dotted line represents the 50% key point division line. **(A,D)** Statistical heatmaps of the GPR and LR models.

We mapped the features of the GPR and LR models with good reliability and validity to the key points of gait (B and C in Figure 8). A dotted line divided 50% of the key points according to their statistics. In the top 50%, the intersection of the two models contained 11 key points, namely, No. 1, No. 3, No. 4, No. 5, No. 6, No. 7, No. 9, No. 10, No. 11, No. 12, and No. 13. We converted the ladder diagram into a heatmap (A and D in Figure 8). The larger the statistical value was, the darker the color, which meant that the corresponding key point had a higher contribution rate to modeling. Except for those of the head and feet, the key points of other body parts presented high heat values.

## Discussion

The results of this study show that personality assessment models based on gait video have good performance in terms of criterion validity, split-half reliability, convergent validity, and discriminant validity. We constructed static features (i.e., gait skeleton coordinates) and dynamic features (including interframe differences, distances between joints, and angles between joints). Interframe differences reflect the changes between different frames, and the distances and angles between joints reflect the states between the joints in the same frame. This gait feature construction method integrating static and dynamic information has been verified in many studies (Pratheepan et al., 2009; Tsuji et al., 2010).

In machine learning, the RMSE is generally used to measure the performance of a model. We found that the mean value of r_1_ was inversely proportional to the mean RMSE value for all models (see Table 4 and Supplementary Appendix A), which shows that it is reasonable to use the criterion validity as an evaluation indicator of model performance. Cronbach’s alpha is the most commonly used reliability statistic. Usually, Cronbach’s alpha is above 0.7, which is considered acceptable reliability (Cronbach, 1951). The alpha coefficient of each trait in BFI-44 is close to 0.8 (Hongyan et al., 2015). For the split-half model containing 74 frames of data, it is impossible to calculate all split-half cases. So we used the odd-even split-half reliability (VandenBos, 2007) approximation as the evaluation index. Except for those of the SVR-poly and RFR models, the mean r_2_ values in each dimension of the other models was higher than 0.8 (see Table 4 and Supplementary Appendix A), indicating that the reliability evaluation of the model was reasonable.

The correlations between the traits in BFI-44 (the lower right italicized areas in Tables 5, 6 and Supplementary Appendix B) showed that extraversion, agreeableness, conscientiousness and openness had positive correlations with each other, and they had negative correlations with neuroticism. A similar pattern was found in the correlations between the dimensions of the GPR and LR models (the upper left italicized area in Tables 5, 6), but no similar patterns were found in the other models. Both reliability and validity studies of the BFI-44 scale (Carciofo et al., 2016) and model-based personality assessment studies (Park et al., 2015) have confirmed the existence of this pattern. Therefore, it has been proven again that the GPR and LR models have good performance in many aspects.

Personality traits are closely related to body movements (Koppensteiner and Grammer, 2010; Thoresen et al., 2012). Some studies have shown that agreeableness and pelvic motion, as well as extraversion and thoracic motion, are positively correlated, and conscientiousness and thoracic motion are negatively correlated (Satchell et al., 2017). This is consistent with our results; that is, Neck (No. 1), RShoulder (No. 2) and LShoulder (No. 5) on the thorax and RHip (No. 9) and LHip (No. 12) on the pelvis all yielded high heat values (Figure 8). In addition, during walking, certain differences are observed between arm swings and leg strides (DeVita et al., 1991). In our results, there were differences between RWrist (No. 4) and LWrist (No. 7) on the hands and between Rankle (No. 11) and LAnkle (No. 14) on the feet (Figure 8). We found that the key points of the head and feet presented low heat values, which might be due to the fact that the changes in these parts (relative changes between internal key points) were not obvious compared with the limbs and trunk, with little individual differences. In previous studies on the relationship between personality and gait (Tolea et al., 2012; Wang et al., 2016; Satchell et al., 2017; Stephan et al., 2018), gait mainly focused on the limbs and trunk with a large range of motion. The above shows that our models learned some of the kinematic characteristics of gait. However, we have only initially explored the interpretability of personality assessment models from one perspective. Further research is needed in the future.

Our research purpose was not to find a machine learning algorithm but to explore the feasibility of predicting personality based on gait video through machine learning modeling and to provide a new idea and method for automatic personality assessment. According to a literature exploration and our knowledge, this study is the first to measure the reliability and validity of machine learning models in the field of automatic personality assessment using gait. In multidimensional studies, measuring the reliability and validity of machine learning models helps to ensure that a model can truly discover the patterns of corresponding traits; this could not be achieved by previous machine learning evaluation methods.

Our study is still in its infancy and cannot completely replace a personality scale, but it has good prospects. Our method needs only 3 s of effective gait video to efficiently and conveniently realize personality assessment. Personality traits are highly stable after adulthood (Cobb-Clark and Schurer, 2012) and are closely related to health statuses (Jokela et al., 2013), occupational tendencies (Kern et al., 2019), and academic achievements (Komarraju et al., 2011). In future job searches, enrollments and other occasions, it will be necessary to make personality assessments for the examinee. By using our method, an evaluation result can be quickly obtained without interference and used to assist the examiner in decision making.

This study exhibits some limitations. First, the educational levels of the participants in the experiment were concentrated in the postgraduate stage, and the relatively concentrated cultural level of the group may have caused their personalities to be similar. Second, deep learning algorithms may improve model performance to a certain extent, but the number of participants in our study was small. Third, we used a single camera to obtain gait videos of participants’ fronts, backs and turns while they walked back and forth. However, only the frontal gait was used in the experiment, which led to data waste. In future studies, we will expand the scope and scale of recruitment, invite participants with large individual differences, and improve our research algorithms and data collection methods.

## Conclusion

This study moves one step forward toward a non-invasive and low-cost personality assessment solution, which will have potential value in personality-related psychological intervention and behavioral decision making. Our experiments show that Big Five personality assessment models based on gait video have good criterion validity, split-half reliability, convergent validity, and discriminant validity. Our preliminary research provides new ideas for evaluating the performance of machine learning models with multidimensional psychological characteristics and points out a possible direction for constructing convenient personality assessment methods.

## Data availability statement

To protect the privacy of the participants, the original datasets in the article cannot be made public. If necessary, feature datasets of gait are available from the corresponding author on reasonable request. Requests to access the datasets should be directed to TZ, tszhu@psych.ac.cn.

## Ethics statement

The studies involving human participants were reviewed and approved by the Institutional Review Board of the Institute of Psychology, Chinese Academy of Sciences (approval number: H15010). The patients/participants provided their written informed consent to participate in this study.

## Author contributions

YW, BL, TZ, and DC contributed to conception and design of the study. TZ collected and provided the data and directed the writing and research process of this manuscript. YW analyzed the data and wrote the first draft of the manuscript. DC and BL proposed improvements to the research method. All authors participated in the editing and reviewing of manuscripts, contributed to the article, and approved the submitted version.
