# Supplementary material for: Reliability and validity analysis of personality assessment model based on gait video
Source: Front Behav Neurosci. 2022 Aug 2;16:901568. doi: 10.3389/fnbeh.2022.901568 (PMC9380895; doi:10.3389/fnbeh.2022.901568)
Supplement: Supplementary file 1 [file Data_Sheet_1.pdf]

Supplementary Material

1 Appendix A

Table A1: Criterion validity and split-half reliability of the RFR personality assessment model.

	$RMSE$	r_1	r_2
Extraversion	5.588	0.235	0.588
Agreeableness	4.964	0.095	0.652
Conscientiousness	6.360	0.166	0.771
Neuroticism	6.686	0.145	0.754
Openness	6.231	0.087	0.733

RMSE, root mean squared error. r_1 represents the criterion validity, and r_2 represents the split-half reliability. All correlation coefficients are highly significant ($p < 0.001$).

Table A2: Criterion validity and split-half reliability of the SVR-linear personality assessment model.

	$RMSE$	r_1	r_2
Extraversion	7.760	-0.109	0.782
Agreeableness	5.608	0.192	0.863
Conscientiousness	8.688	-0.115	0.849
Neuroticism	8.334	0.071	0.805
Openness	6.865	0.198	0.801

RMSE, root mean squared error. r_1 represents the criterion validity, and r_2 represents the split-half reliability. All correlation coefficients are highly significant ($p < 0.001$).

Table A3: Criterion validity and split-half reliability of the SVR-poly personality assessment model.

	$RMSE$	r_1	r_2
Extraversion	5.609	0.297	-0.290
Agreeableness	4.808	0.255	-0.185
Conscientiousness	6.188	0.274	0.845

Neuroticism	6.526	0.258	0.802
Openness	5.882	0.346	0.803

RMSE, root mean squared error. r_1 represents the criterion validity, and r_2 represents the split-half reliability. All correlation coefficients are highly significant ($p < 0.001$).

Table A4: Criterion validity and split-half reliability of the SVR-rbf personality assessment model.

	$RMSE$	r_1	r_2
Extraversion	5.535	0.332	0.892
Agreeableness	4.707	0.364	0.922
Conscientiousness	6.331	0.214	0.894
Neuroticism	6.517	0.315	0.873
Openness	5.872	0.442	0.921

RMSE, root mean squared error. r_1 represents the criterion validity, and r_2 represents the split-half reliability. All correlation coefficients are highly significant ($p < 0.001$).

Table A5: Criterion validity and split-half reliability of the SVR-sigmoid personality assessment model.

	$RMSE$	r_1	r_2
Extraversion	5.606	0.225	0.749
Agreeableness	4.658	0.328	0.925
Conscientiousness	6.256	0.237	0.981
Neuroticism	6.402	0.317	0.906
Openness	5.679	0.473	0.917

RMSE, root mean squared error. r_1 represents the criterion validity, and r_2 represents the split-half reliability. All correlation coefficients are highly significant ($p < 0.001$).

2 Appendix B

Table B1: Convergent and discriminant validity of the RFR personality assessment model (RFR-PAM).

	RFR-PAM					BFI-44				
	E	A	C	N	O	E	A	C	N	O
RFR-PAM										
E										
A	*-0.004*									
C	*0.031*	*0.003*								
N	*-0.071*	*0.015*	*-0.271*							
O	*0.048*	*0.048*	*0.141*	*-0.055*						
BFI-44										
E	**0.235**	0.006	-0.014	-0.014	-0.011					
A	0.101	**0.095**	-0.006	-0.016	-0.038	*0.415*				
C	0.146	0.056	**0.166**	-0.053	-0.029	*0.454*	*0.523*			
N	-0.101	0.005	-0.068	**0.145**	-0.019	*-0.596*	*-0.678*	*-0.584*		
O	0.124	0.134	0.027	-0.038	**0.087**	*0.273*	*0.220*	*0.448*	*-0.285*	

E, extraversion; A, agreeableness; C, conscientiousness; N, neuroticism; O, openness. All correlation coefficients are highly significant ($p < 0.001$).

Table B2: Convergent and discriminant validity of the SVR-linear personality assessment model (SVRlinear-PAM).

	SVRlinear-PAM					BFI-44				
	E	A	C	N	O	E	A	C	N	O
SVRlinear-PAM										
E										
A	*0.020*									
C	*0.055*	*-0.023*								
N	*-0.084*	*-0.016*	*-0.104*							
O	*-0.050*	*-0.065*	*-0.056*	*-0.023*						
BFI-44										
E	**-0.109**	-0.027	-0.062	-0.016	0.101					

A	-0.146	**0.192**	-0.091	0.012	0.040	*0.415*				
C	-0.053	0.169	**-0.115**	-0.073	0.089	*0.454*	*0.523*			
N	0.106	-0.069	0.034	**0.071**	-0.052	*-0.596*	*-0.678*	*-0.584*		
O	0.046	0.086	0.044	-0.081	**0.198**	*0.273*	*0.220*	*0.448*	*-0.285*	

E, extraversion; A, agreeableness; C, conscientiousness; N, neuroticism; O, openness. All correlation coefficients are highly significant ($p < 0.001$).

Table B3: Convergent and discriminant validity of the SVR-poly personality assessment model (SVRpoly-PAM).

	SVRpoly-PAM					BFI-44				
	E	A	C	N	O	E	A	C	N	O
SVRpoly-PAM										
E										
A	*0.053*									
C	*0.101*	*0.005*								
N	*0.036*	*0.030*	*-0.145*							
O	*0.129*	*-0.071*	*0.040*	*-0.316*						
BFI-44										
E	**0.297**	0.090	0.111	-0.066	0.093					
A	0.170	**0.255**	0.258	-0.162	0.130	*0.415*				
C	0.085	0.229	**0.274**	-0.063	0.093	*0.454*	*0.523*			
N	-0.127	-0.225	-0.238	**0.258**	-0.152	*-0.596*	*-0.678*	*-0.584*		
O	0.140	0.097	0.107	-0.133	**0.346**	*0.273*	*0.220*	*0.448*	*-0.285*	

E, extraversion; A, agreeableness; C, conscientiousness; N, neuroticism; O, openness. All correlation coefficients are highly significant ($p < 0.001$).

Table B4: Convergent and discriminant validity of the SVR-rbf personality assessment model (SVRrbf-PAM).

	SVRrbf-PAM					BFI-44				
	E	A	C	N	O	E	A	C	N	O
SVRrbf-PAM										

E										
A	*0.167*									
C	*0.323*	*0.267*								
N	*-0.340*	*-0.222*	*-0.150*							
O	*0.061*	*0.050*	*0.016*	*0.044*						
BFI-44										
E	**0.332**	0.049	0.154	-0.259	0.168					
A	0.171	**0.364**	0.175	-0.217	0.107	*0.415*				
C	0.231	0.173	**0.214**	-0.225	0.189	*0.454*	*0.523*			
N	-0.140	-0.187	-0.090	**0.315**	-0.113	*-0.596*	*-0.678*	*-0.584*		
O	0.149	0.116	0.081	-0.153	**0.442**	*0.273*	*0.220*	*0.448*	*-0.285*	

E, extraversion; A, agreeableness; C, conscientiousness; N, neuroticism; O, openness. All correlation coefficients are highly significant ($p < 0.001$).

Table B5: Convergent and discriminant validity of the SVR-sigmoid personality assessment model (SVRsigmoid-PAM).

	SVRsigmoid-PAM					BFI-44				
	E	A	C	N	O	E	A	C	N	O
SVRsigmoid-PAM										
E										
A	*0.043*									
C	*0.072*	*-0.097*								
N	*-0.213*	*0.006*	*-0.172*							
O	*0.036*	*0.295*	*-0.013*	*0.031*						
BFI-44										
E	**0.225**	0.036	0.082	-0.181	0.101					
A	0.054	**0.328**	0.171	-0.252	0.140	*0.415*				
C	0.171	0.211	**0.237**	-0.165	0.209	*0.454*	*0.523*			
N	-0.144	-0.283	-0.176	**0.317**	-0.111	*-0.596*	*-0.678*	*-0.584*		
O	0.086	0.269	0.102	-0.157	**0.473**	*0.273*	*0.220*	*0.448*	*-0.285*	

E, extraversion; A, agreeableness; C, conscientiousness; N, neuroticism; O, openness. All correlation coefficients are highly significant ($p < 0.001$).
